# Covid-19 and mobile payment in Belgium: Closing the digital divide or just for the young, social, and impulsive?

**DOI:** 10.1007/s10660-022-09655-4

**Published:** 2022-12-28

**Authors:** Johan Hellemans, Kim Willems, Malaika Brengman

**Affiliations:** Department of Business, Marketing & Consumer Behavior Cluster, Imec-SMIT, Vrije Universiteit Brussel, Pleinlaan 11, 1050 Brussels, Belgium

**Keywords:** Mobile payment, Social network sites, Impulse buying tendency, Convenience stores, Digital divide

## Abstract

Experts and industry reports agree that the COVID-19 crisis spurred the adoption of new retail technologies, like mobile payment. However, empirical academic evidence that compares their adoption and usage before, during, and after the crisis remains scarce. So far, academic mobile payment research has focussed almost entirely on the different building blocks of technological acceptance models, like perceived usefulness and ease of use, and their role in explaining intention to use. We need to learn more about the profile of the actual user. In this Belgian study, we investigate the evolution in mobile adoption based on survey data from 2019 to 2020 (2019: N = 897; 2020: N = 895). We examine differences in the profile of mobile payers in terms of their socio-demographics, retail, and social media behaviours. The pandemic triggered a clear uplift in mobile payment users between 2019 to 2020. Nonetheless, striking differences in socio-demographic profile and retail patronage remain. Our data shows that there is still inequality in adoption, related to age and social grade. We also observe a clear association between general impulse buying tendency and mobile payment. The link between internet/online shopping and mobile payment is firmly established. Finally, mobile adoption is related to the use of Instagram and Facebook. Consequences for retailers, researchers and public officers are further discussed.

## Introduction

Just 20 years ago, the internet was, at best, a non-compulsory distribution, transaction, and communication channel [1]. Now many see a decline in brick-and-mortar retail due to the proliferation of online shopping and e-commerce as inevitable [2]. Against this trend, integrating and harmonizing innovative technologies, such as mobile payment, are crucial to enable an omnichannel strategy offline retailers can deploy to ensure a better customer experience [3–5]. Customers seek speed, convenience, and flexibility with every purchase, regardless of the retail channel [6]. Whereas online and offline retail were still two distinct worlds in the past, the two are rapidly converging, thanks to mobile e-commerce, which has been seen as a primary facilitator in the process [7]. Ultimately, the payment transaction is the component that completes, delays, or even defers the purchase phase. Thus, mobile payment technology is important to retailers, as it facilitates and encourages the finalization of sales transactions. Research shows that, especially for online purchases, much hesitation can occur in the final payment phase and ultimately cause the purchase process to be aborted and the shopping cart to be abandoned [8]. However, offline retailers are also expected to provide easy and secure payment, as the perception of offline purchases with a payment technology positively impacts corresponding online usage [9]. Furthermore, research has demonstrated that mobile payment leads to a higher willingness to purchase and pay because of its low pain and high convenience [10]. Such research indicates that mobile payment stimulates consumption. There is little doubt that secure, easy, and smooth payment is a crucial lever to improve and finalize not only the transaction but also benefits the overall customer's experience. Mobile payment has changed consumers' spending patterns, as transactions can be made anytime, anywhere [11]. Payment via digital payment is considered more convenient, easier, and quicker than the use of traditional technologies such as credit cards, checks, or cash [12].

Mobile payment can be defined as any system that offers service of financial activities, which includes the initiation, activation, and confirmation (authentication and authorization) of payment, either online or offline, using a mobile device, through different technology, applications, and solutions provided by banks or proxy financial companies [10, 12–15].

With the speed of growth of financial technology, so-called fintech, and its introduction into the market, research investigating the mobile payment, as a specific subtype of fintech, attracted significant attention since technology made transactions via a mobile device possible. Especially since the corona crisis, the adoption of innovative technologies in retail has accelerated even further [17, 18]. Even so, mobile payment remains a new domain of research. With the rapid rise of digital technology over the last decades, academic research is lagging, with many questions left unanswered [16].

This study aims to answer the following questions, which we elaborate on in the subsequent sections: (1) How did the adoption of mobile payment change over the corona-crisis? (2) Which consumer groups have adopted mobile payment, and which have not? (3) Which individual differences and retail contexts beyond demographics might explain adoption further? (4) what is the role of social media in the adoption process?

A better understanding of these questions enables policymakers to understand which target groups to focus on for marketing and communication strategies, mainly social media. Thus, contributing to more efficient and effective policies toward financial inclusion. For retailers, such insight supports better decision-making on which payment systems to offer. Given the evolution towards a cashless society, addressing these questions gives all actors levers to ensure everyone can use reliable and convenient payment systems.

This study adds to ongoing research on technological applications during the pandemic. Experts agree that the COVID-19 crisis accelerated the adoption of certain online and traditional retail technologies [18]. In a pandemic world where contactless interaction is advocated, it can be expected that contactless payment thrives even more than before to limit in-person transactions. However, little empirical evidence has been found comparing the adoption and usage of mobile payment technology before, during, and after the crisis. So, our first research objective is to investigate to which degree mobile payments were adopted, comparing data from 2019 and 2020.

Understanding how consumers differ in their purchase behavior in a multichannel context becomes crucial [19]. It is more important than ever for retailers to understand their customer demographic profiles when adopting recent technologies [15]. However, researchers studying mobile payment have focused entirely on technological acceptance models and which of those models' theoretical mediating constructs are most likely to influence intention to adopt mobile payment services [11, 20, 21]. Such models provide a clear view of the criteria the technology must meet to impact intention and provide input to developers in understanding which benefits consumers seek and what characteristics the technology must possess to be relevant in the marketplace. However, they offer little guidance to retailers in understanding the profile and number of users capable of using specific technology, in which context it can best be applied, how to promote it, and to whom. The mobile payment field is built on explaining the intention to use, making no difference between actual users and nonusers. As a result, little is known about the current real users of mobile payments [11]. While investigating socio-demographic differences between users and nonusers in other technology domains like social media [22] or mobile shopping [23] is a frequent practice, such insights seem notably lacking when investigating the literature on the adoption of mobile payment technologies. Recent work in financial policy research tried to identify the [early] adopters of mobile payment, but also, in that area, such research remains limited [12, 24, 25]. Earlier work advised the inclusion of contextual factors indicating that mobile payment needed to fit into the life of its users [26], and mobile payment had certain advantages depending on specific situations [27]. The significance of adoption factors in different situations like the type of shop [e.g., supermarkets, kiosks, vending…] and various payment scenarios in terms of technology applied at the points of sale require further investigation [21].

The second objective of this paper is to contribute to the mobile payment research field by addressing these gaps. By investigating differences in the profile of mobile payers in terms of their socio-demographics, frequency of patronizing convenience retail channels, social media usage, and their underlying general impulsive buying tendency, we provide insight into the mobile payment acceptance research domain in selecting potential essential moderators and context factors. Moreover, considering the continuous advancement of digital innovations, their importance, and their penetration into daily life, such research can also inform retailers, developers, and policymakers, on which digital/technology strategy to follow, making a segmented marketing and communication strategy possible [28].

Digital inequality and the "digital divide" concept refer to differences in digital access, information, tools, and usage between regions and people. Earlier work within the digital divide field focused on access to computers, the internet, and the significance of having access when and where one wants it [29]. In developed countries where access to the internet is seen as standard and in reach for most people regardless of urbanization, education, and age, the focus has shifted towards specific access to certain digital tools and usages like social media or mobile payment. In the related field of financial inclusion, the impact of fintech is studied to enable better access to financial services [30]. It shows that investigating socio-demographic factors as antecedents of mobile payment adoption has merit.

Mobile payment solutions have found their way into both developed and developing countries [31], but the perceived benefits of such technology between cultures are different [32]. In emerging markets, financial technology can secure financial inclusion for groups that would not otherwise have access to financial services. [31]. For developing countries, financial inclusion and better access to financial services are crucial for improving the lives of their citizens [30]. Recent work in China suggests a positive association between mobile payment usage and happiness [33]. As such, ensuring more mobile banking and payment adoption is recommended strongly to policymakers of emerging countries [34]. It might also explain why research in the mobile payment field seems skewed towards developing and emerging countries, and evidence in the context of advanced economies is limited [14, 20]. In contrast, mobile payment adoption in developed countries has been associated with increased financial vulnerability. This finding is supported by research in the US while not confirmed by recent work in a European context [12]. Given such differences, new legislation, new technologies, and the market growth within mobile payment, investigating which groups are most likely to adopt digital payment and their consequences remain a constant endeavour. There is a definite need for more research from different countries [12]. We report data from Belgium based on a representative non-student sample and enrich the field from a cultural perspective. We report data from Belgium based on a representative non-student sample and enrich the field from a cultural perspective. Cash still accounts for 73% of payment transactions and 48% in value within the Eurozone [35]. so even in developed countries, the technology still needs to be widely accepted and used. Belgium is an interesting case to research the adoption of mobile payment given that pre-crisis adoption of contactless and mobile payments was lower than in other Eurozone countries [36].

Given that recent review articles have been published that give an update on the status of the research on mobile payment, this paper focuses on discussing relevant studies related to our research questions that provide arguments in support of our hypotheses. An exhaustive analysis of all research on mobile payment is not within the scope of the current paper. In the next section, we discuss successively; (1) the role of the covid-19 crisis, (2) socio-demographic determinants, (3) shop patronage and general impulse buying as retail determinants, and (4) social media determinants. Next, we elaborate on the methodology and detail the findings of our study. The conclusion of the final section includes limitations, managerial implications, and directions for future research.

## Literature

### Adoption of Mobile payment and the covid-19 crisis

Even before COVID-19, retailing was dramatically spurred by recent technologies [37], including mobile (contactless) payment [7]. While in Europe, mobile banking grew slower [14], it is hypothesized that the COVID-19 pandemic accelerated the adoption of digital payments among customers and retailers [20, 37].

The COVID-19 crisis impacted retailers severely, making physical shop activities hard to impossible due to widespread restrictions [social distancing, lockdowns, quarantines, business closures]. Whereas in normal circumstances, digital technology and its development already profoundly impacted consumer behavior in the last decades, the recent COVID-19 crisis made them indispensable for many aspects of daily functioning [38]. Technological advancement has enabled companies to continue running throughout the crisis [6]. Some consumers, for instance, still preferred the in-store purchase method, and mobile payment was seen as a contactless safer option to enable such choice [39]. Other consumers moved to online shopping, home deliveries, or cashless payment, which they had never considered before [40].

With the rapid and sudden changes in the use of digital devices and services, researchers must also consider the extent to which trends and research findings from the past still apply and are relevant today [41]. The factors that matter in an emerging market may not be the same once it transitions into a mature market. While traditionally, the primary device for mobile payment were smartphones, payment with wearable technology, still in the beginning stages of its product life cycle, might become more common and offer further benefits toward a cashless society [42]. In addition, research should establish to which degree findings obtained during the COVID-19 pandemic are relevant once restrictions are lifted. Findings from Germany reveal that while certain consumers adopted online grocery purchases, most consumers returned to offline grocery purchases after pandemic restrictions were lifted [43]. In mobile payment, most researchers expect its usage to increase further. However, some researchers believe security concerns and risks experienced by new users of mobile payments amidst COVID-19 may hinder further use or adoption if not mitigated [44].

There is undoubtedly a need for retail managers and marketers to monitor the changes in consumers' shopping behavior and which changes and habits are here to stay, to understand which changes in strategies they need to adopt, and to anticipate what the landscape for retailers will be after the pandemic [38, 45].

Mobile payment services are expected to grow in double digits [10]. Scholars also argue that proximity payment companies like Apple and Google pay provide more opportunities for customers to adopt mobile payments [14], so we expect this trend to continue and the number of adopters and regular users to increase further.

#### H1

The number of users adopting mobile payment significantly increased in 2020 compared to 2019.

### Mobile payment adoption

Researchers interested in the field of mobile payments mainly rely on one or more competing IT adoption models like the technology acceptance model [TAM] of Davis [46], the unified theory of acceptance and use of technology UTAUT by Venkatesh et al. [47], its successor UTAUT2 [48] to explain intention to use mobile payment technology. Several reviews and meta-analyses shed light on which of those models' theoretical constructs are most likely to influence the intention to adopt mobile payment services [11, 20, 21, 49]. Researchers have extended or integrated many building blocks of these models, which results in numerous factors in Mobile payment acceptance research [13]. While the intention to use a specific technology was the primary variable of interest [11], some researchers stressed the importance of actual use and future continuity [50, 51], and earlier work already indicated differences among users and nonusers [52].

According to a recent review of 25 research within the mobile payment field that has extended the UTAUT model, only perceived risk, perceived trust, perceived cost, and self-efficacy showed consistent significant association within the UTAUT model among no less than 46 factors. The best predictors of intention to use mobile payment are performance expectancy, social influence, effort expectancy, perceived trust, perceived cost, and self-efficacy [13]. A meta-analysis of sixty-one papers published between 2008–2017 shows that perceived usefulness, perceived risk, social influence, trust, and perceived ease of use significantly explain consumers' intention to use mobile payment [11]. Most of the key constructs in those models relate to universal and specific needs and benefits consumers seek, either related to a utilitarian or hedonic performance expectancy, followed by convenience and social influence [53]. Such models provide a clear view into the criteria the technology must meet to impact intention and provide input to developers in understanding which benefits consumers seek and what characteristics the technology must possess to be relevant in the marketplace. However, while these factors are known, little to no research exists that investigates the antecedents of adoption factors, what they mean, in which situation they apply, by whom, and how they can be changed and managed [21]. Furthermore, Dahlberg and colleagues concluded that in more recent literature, the same findings were put forward as in earlier contributions. The research field shows little progression due to an overly one-sided approach to the topic [21, 51]. Reviewing research applying the extended Unified Theory of Acceptance and Use of Technology suggests that researchers seldom include moderators. At the same time, the model implicitly foresees age, gender, and experience as user class moderators [54]. Meta-analysis of research using the UTAUT model did not include moderators because, according to the authors, prior studies had not examined those moderators or not reported information about those moderators [55]. Even if some socio-demographic factors are included as moderators [9, 56–59], they do not offer insight into actual differences in adoption or usage. For instance, even if gender or age moderate a factor in a technology acceptance model, it does not necessarily imply higher or lower adoption or usage [56]. Most scholars agree that consumers' characteristics also play a vital role in adopting mobile shopping or payment technology. However, the research that profiles users and provides such characterization is lacking [53].

#### Socio-demographic factors as determinants of mobile payment adoption

Given that the research domain ignored socio-demographic indicators, we cannot formulate a priori hypotheses about the role of individual differences based on the technology acceptance research of mobile payment. Therefore, we relied on adjacent research conducted in the financial policy domain that tried to identify and profile the adopters of mobile payment for policy reasons [12, 24, 25]

In a study involving more than 25,000 US households, Meyll and Walter [60] report use of mobile payment is more pronounced among the younger population and men. Furthermore, mobile payment users tend to be more likely unmarried and childless. They also have obtained a higher level of education and income. No difference was found in their occupational status. An earlier study in the US, using a large national representative sample of 15,060 households, found that the profile of mobile payment users differed significantly from nonusers. They also reported a higher incidence of mobile payment adoption for younger age groups and men. Also, higher education and income showed higher odds for mobile payment adoption [61]. Other research using an online national survey interviewing 1497 American respondents did not support the higher odds for higher education and income nor their working or marital status. However, age and being male were significant in explaining the adoption of mobile financial services [62]. An online survey of 937 mobile shoppers in the US found that education and income levels significantly increased mobile shopping intensity purchases. Also, males indicated spending more on mobile shopping than females, and younger ages also made more mobile purchases [23]. Based on a survey amongst 323 German households, the research found a negative association between increasing age and intention for switching to a fintech, so a higher affinity for digital innovations for younger age groups. Gender, civil state, children in the household, nor an aggregated social grade factor based on income, education, and employment showed a significant difference in their model [25].

A recent study collected data from a representative sample of the Norwegian adult population (n = 2202) to investigate individual differences in the adoption of online, mobile, and contactless payment and the willingness to use social media for money transfers. The study shows that women report a higher incidence of online and mobile payments but a lower odd of using social media companies for money transfers. The effect of age is apparent. Younger generations show higher odds for all three digital payment methods and the willingness to use a social media company for money transfers. Furthermore, also higher income is associated with more mobile and contactless payment, whereas for higher education, this applies to online payment only [12]. A previous survey in Finland amongst 2675 customers of a Scandinavian bank indicated that mid-aged individuals significantly adopted mobile banking more than the youngest age groups, and men reported higher odds for mobile banking. Education, income, occupation, and household size did not show a significant effect [63].

Two major international barometers monitoring financial inclusion shed further light on mobile payments in Belgium [35, 64]. The current data reported in both studies do not allow us to distinguish mobile payment overall. However, the World Bank reports that 33% of the adult population above 15 years of age made a digital in-store payment using a mobile phone in 2021. The reported mobile in-store payment is higher for men than women (36% versus 30%). They further differentiate between ages, with 15–24-year-olds reporting higher use (38%) than those older than 24 (32%). They also noted a lower incidence for those with a primary education only compared to those with at least a secondary or higher education (14% versus 34%) [64]. The European central bank details E-payment solutions for online purchases. Belgium is ranked 15th out of 19 countries, with E-payment being 20% of the total transactions of this type, representing 17% in value in 2019. Both are lower than the average reported across countries, with 27% of the transactions being E-payment representing 24% of the online value [35]. These studies further show that Eurozone countries still have significant differences in payment behavior. Differences in digital payment behavior between population groups depend on income, education, age groups, and the type of purchase [35, 64]. Research of Febelfin, an organization representing financial institutions in Belgium, reported on their website that 4 in 10 Belgians use mobile payments in 2022, with 33% of Belgians saying they have already paid via a QR code in a shop and 21% via a wearable. In their survey, mobile payment is the most popular among younger generations. They expect mobile payment adoption to rise further. From 1 July 2022, merchants are legally obliged to offer digital payment methods [65]

While the above literature is not conclusive, we tend to hypothesize the following based on these previous findings:

##### H2

Mobile payment adoption is higher among men compared to women.

##### H3

Mobile payment adoption is higher among younger age groups compared to older age groups.

##### H4

Mobile payment adoption is higher among singles compared to those living together.

##### H5

Mobile payment adoption is higher among those without children compared to those with children.

##### H6

Mobile payment adoption is higher among better socioeconomic groups compared to lower socioeconomic groups.

##### H7

Mobile payment adoption is higher among higher income groups compared to lower income groups.

#### General impulsive buying tendency and convenience store patronage as determinants of mobile payment adoption

From early work onwards, it has been hypothesized that the use of mobile payment could be specific to shopping situations and outlets [26, 27]. Also, Dahlberg et al. recommended including contextual factors in investigating different retail and shopping scenarios [21].

Recent studies have explored the relationship between mobile payment and impulse buying intention. They provide evidence that the mobile shopping environment is a context that can trigger enjoyment and arousal that leads to more impulsive buying. [66–68]. Increased consumer impulse spending is mentioned as one of the benefits for retailers to adopt mobile payments, and it is assumed that impulse purchases will increase with the adoption of mobile payments [69]. Research also shows that motivations like convenience, bargain hunting, and enjoyment related to a more impulsive shopping style are related to the number, frequency, and money spent on mobile shopping [23]. The use of mobile payments also increases the willingness to pay [10] and is assumed to lead to an additional decrease in spending control [12]. Mobile payment users might also be prone to impulse spending [61].

In the current research, we conceptualize buying impulsiveness as a trait [70, 71], with some individuals showing a higher tendency to buy on impulse and others showing a lower tendency [72, 73]. Given the findings above, we hypothesize that general impulsive buying tendency is associated with mobile payment adoption, given its correlation with impulse buying behavior.

##### H8

Mobile payment adoption is higher among individuals with a higher general impulse tendency.

It has been postulated that mobile payment is exciting and adopted if consumers perceive a fit and need within their lifestyle [27]. We assume this to be higher amongst online shoppers and visitors of convenience stores like night shops, petrol stations, and vending machines, given that these channels are more oriented towards convenience and impulse.

Given that mobile payments are associated with convenience, we hypothesise mobile payments are more frequent among those shopping online or patronaging convenience channels.

##### H9

Mobile payment adoption is higher among online shoppers compared to non-online shoppers.

##### H10

Mobile payment adoption is higher among visitors to convenience stores compared to those who do not visit such stores.

#### Social media usage as determinant of mobile payment adoption

Social media is omnipresent and has changed almost any aspect of society, facilitating, and transforming information and communication [74]. Social network sites can be seen as particular instances of an organizational class factor within the technology acceptance literature that offers social context for adopting technology [54] that, given its pervasiveness, might have become more critical. Within the research applying technology acceptance models, there is a clear indication that social influence, subjective norms, or social norms are influential factors in predicting the intention to adopt mobile payment [47]. This finding is often why such research advocates social media marketing campaigns to steer mobile payment adoption [43]. During the COVID- 19 pandemic, individuals also relied on information commonly shared over social media networks [75], including normative messages about which behavior was appropriate, with mobile payment as a safer payment choice. Hence, we hypothesize that users of social network sites would adopt mobile payment more than those that do not use such platforms as they seek appropriateness of behavior.

##### H11

Mobile payment adoption is higher among social media users than those who do not use it (regularly).

## Methodology

### Sampling

We received repeated cross-sectional data from a Belgian online panel survey that probed for the same information at two successive time points resulting in two independent samples, one for 2019 and one for 2020. All data received was gathered, anonymized, and deidentified before access in compliance with general data protection regulation (GDPR). Quotas were imposed on age, gender, and region to obtain similar sample structures between 2019 and 2020. The interviews were controlled for the interview's duration, answers' completeness, and response style. Speedsters, incomplete interviews, and similar response styles across all questions were deleted. Potential Common Method Variance (CMV) bias was addressed a priori at the questionnaire set-up [76],. Harman's single-Factor Test was conducted, with the first unrotated factor accounting for only 28% of the variance, below the threshold of 50% suggested in this approach.

The datasets generated and analysed during the current study are not publicly available because they constitute an excerpt of research in progress and are based on proprietary company information, we received from a third-party market research company.

Without being exhaustive, we would like to outline the period during which the samples were realized briefly. The number of corona measures implemented in Belgium is diverse and extensive. The data for 2019 was obtained in January 2020 and preceded the period that Belgium went into lockdown. From mid-March, all non-essential stores closed, all gatherings prohibited, and limited social contact outside the family was installed. From April onwards, a series of gradual relaxations were implemented. From October, Belgium's government gradually tightened its measures again, resulting in a new lockdown phase beginning of November that will extend from 2020 to 2021. The second data for 2020 was obtained during that period in January 2021.

We obtained a sample of 1792 observations (2019: N = 897, avg age = 44.5, SD = 13.04, of which 49% were woman; 2020: N = 895, avg age = 44.1, SD = 13.50, of which 49% were woman; 2021). Quota sampling methods were employed regarding age, sex, and region. The sample description can be observed in Table 1. We further checked for underlying socio-demographic differences per profile between both years. Table 1 shows the samples' profiles are similar, based on Pearson chi-square tests (year*(socio-demographic variable)), conducted with none showing any significant difference.

**Table 1:** Sample description

	Sample size	2019	2020	Total	Chi-Square Tests
		897	895	1792	
Region	Flanders	59%	56%	58%	χ2 = 1.30,df = 1, p = 0.25
	Walloons	41%	44%	43%	
Urban Area	No	46%	45%	46%	χ2 = 0.58,df = 1, p = 0.45
	Yes	54%	55%	55%	
Gender	Man	51%	51%	51%	χ2 = 0.08,df = 1, p = 0.78
	Woman	49%	49%	49%	
Age	18–25	12%	13%	12%	χ2 = 1.05,df = 4, p = 0.90
	26–35	16%	17%	16%	
	36–45	22%	21%	22%	
	46–55	26%	24%	25%	
	56–65	25%	26%	25%	
Single	No	61%	61%	61%	χ2 = 0.08,df = 1, p = 0.78
	Yes	39%	39%	39%	
Kids	No	45%	46%	45%	χ2 = 0.02,df = 1, p = 0.89
	Yes	55%	55%	55%	
Education level	Lower	12%	11%	12%	χ2 = 3.77,df = 3, p = 0.29
	High School	42%	46%	44%	
	Graduate	22%	20%	21%	
	Master	23%	24%	24%	
Income class	-30 k	33%	29%	31%	χ2 = 3.85,df = 3, p = 0.28
	30-50 k	38%	39%	39%	
	50-70 K	19%	20%	20%	
	70 k +	10%	11%	10%	
Working	No	40%	37%	39%	χ2 = 1.74,df = 1, p = 0.19
	Yes	60%	63%	61%	
Occupation	Independent	4%	3%	3%	χ2 = 4.60,df = 6, p = 0.60
	Managerial	9%	10%	9%	
	White-collar	19%	20%	20%	
	Blue-collar	28%	29%	29%	
	Unemployed	7%	7%	7%	
	At home	27%	24%	25%	
	Student	6%	6%	6%	

### Socio-demographic profile measures

Besides age and gender, the survey probed for other socio-demographic variables. The following categorical variables were obtained: (1) education level, (2) professional/occupational situation, (3) civil state, (4) household income, (5) children in the household, (6) region (French-speaking part versus Flemish-speaking part), and (7) urban area.

Given that responses about socioeconomic characteristics, such as education, employment status, and income, can show a high level of association and relate to other demographic variables like age, they are also often grouped in a social grade composite score (SGSS) constructed from socioeconomic characteristics. In our case, the variable differentiates seven groups from score one, meaning lower, to seven, indicating a higher social milieu, similar to Esomar [77] and other academics [25].

### Shopper profile measures

Respondents indicated whether to be regular shoppers of (1) online shopping and convenience stores like (2) vending machines, (3) night stores, (4) gas stations, and (5) newspapers kiosks.

General impulsive buying tendency (GIBT) was measured using an adapted version of Weun, Jones, and Beatty [72]. Respondents rated a 6-point scale going from (1) never to (6) always on the following items; (1) I am a person who makes unplanned purchases, (2) When I go shopping, I buy things that I had not intended to purchase, (3) I enjoy buying something spontaneously, (4) If I come across interesting promotions, I am inclined to buy them without much overthinking, (5) When shopping I look around for promotions I had not foreseen, and (6) I take notice of what might be in promotion, to score an unforeseen deal. Exploratory factor analysis showed one factor explaining 67% of the variance. Cronbach's alpha for both the Dutch version [Mean = 13.57, SD = 6.83, α = 0.91] and the French version [Mean = 14.47, SD = 7.05, α = 0.92] showed adequate reliability.

### Media usage profile measures

Data on respondents (social) media access were obtained for the following media channels and platforms: (1) Facebook, (2) Twitter, (3) Instagram, and (4) YouTube, as well as (5) internet browsing, (6) digital newspapers, (7) digital magazines, (8) TV, (9) radio, (10) newspapers, and (11) magazines.

Daily active users (DAU), defined as users that go on their social media at least once a day, were attained for all media. The DAU measure is a standard dichotomized metric used by social network platforms and industry press reports and used in other research investigating the adoption of social network sites [78–80]. The same measurement was applied to the other media.

### Mobile payment adoption measures

Finally, participants rated how frequently they have used mobile (contactless) payment (apps) over the last 12 months on a single 7-point frequency scale, going from "not at all" (i.e., score 1) to "Once a week or more" (i.e., score 7) in a similar vein like Pal, Herath, and Rao [50]. Our definition of mobile payments includes any payment made with a mobile device using SMS, QR, App, or wallet.

## Results

### Mobile payment

The measure was dichotomized for further analysis, given its skewness to the right, resulting in a non-normal distribution of the mobile payment user frequencies, which is common in profiling users [23–25, 60–63]. We differentiated between regular mobile payment (RMP), defined as at least once a month (1 = scores 5 to 7) and non-regulars (0 = scores 1 to 4), and adoption of mobile payment (AMP) specified has tried it at least once during the year (1 = scores 2 to7) versus nonusers (0 = score 1). The results will be further analyzed with non-parametric statistical testing.

The total sample and the univariate relationship between socio-demographic profiles and AMP and RMP are reported in Table 2.

**Table 2:** RMP and AMP prevalence rates per year and sociodemographic profile

	Total	AMP%			RMP%		
		2019	2020	Total	2019	2020	Total
		897	895	1792	897	895	1792
		26%	46%	36%	20%	37%	28%
Region	FL	27%	50%*	38%	20%	39%*	29%
	FR	24%	41%*	33%	20%	35%*	28%
Urban	No	24%	46%*	35%	19%	35%*	27%
	Yes	27%	46%*	37%	21%	39%*	30%
Sex	Male	28%	48%*	38%	21%	38%*	29%
	Female	24%	44%*	34%	18%	36%*	27%
Age	18–25	55%	67%*	61%	42%	53%*	48%
	26–35	40%	65%*	52%	31%	54%*	43%
	36–45	27%	51%*	38%	19%	45%*	32%
	46–55	18%	39%*	28%	14%	31%*	22%
	56–65	11%	26%*	19%	9%	18%*	13%
Being Single	No	24%	46%*	35%	19%	38%*	28%
	Yes	29%	46%*	38%	21%	36%*	29%
Kids	No	20%	38%*	29%	14%	30%*	22%
	Yes	31%	53%*	42%	24%	43%*	34%
Education	Lower	17%	42%*	29%	14%	31%*	22%
	High school	25%	44%*	35%	17%	35%*	27%
	Graduate	22%	46%*	33%	18%	39%*	28%
	Master	36%	53%*	44%	29%	42%*	35%
Income	-30 k	19%	41%*	30%	14%	35%*	24%
	30-50 k	27%	44%*	36%	20%	35%*	28%
	50-70 K	31%	50%*	41%	25%	39%*	32%
	70 k +	37%	58%*	48%	27%	46%*	37%
Working	No	22%	34%*	28%	17%	27%*	22%
	Yes	29%	53%*	41%	21%	43%*	33%
Professional	Independent	33%	46%*	39%	27%	43%*	34%
	Managerial	38%	67%*	54%	27%	54%*	42%
	White-collar	30%	58%*	44%	23%	46%*	35%
	Blue-collar	25%	45%*	35%	18%	37%*	27%
	Unemployed	25%	27%*	26%	24%	23%*	23%
	At home	14%	28%*	21%	11%	22%*	16%
	Student	52%	65%*	59%	39%	53%*	46%
Professional	White-collar	33%	60%*	47%	24%	49%*	37%
Grouped	Blue-collar	25%	45%*	35%	18%	37%*	28%
	Non-working	22%	34%*	28%	17%	27%*	22%

Chi-Square Tests 2020–2019*p < 0.05

Almost half (46%) of the adult population, 18 to 65 years old, reported having at least tried mobile payment in 2020. A significant increase from the 26% reported in 2019 [χ2 = 78.24, p = 0.00]. A similar trend for the RMP score can be noted. The RMP% increases from 20% in 2019 to 37% in 2020 [χ2 = 66.41, p =  < ,001].

The significant chi-squares values for most group*period cross-tabulations in Tables 2 and 3 indicate that overall, the RMP and AMP prevalence of mobile payment increased significantly during the corona crisis amongst almost all groups under investigation. Exceptions are the AMP scores for the youngest age group, students, and independent professionals showing no significant increase.

**Table 3:** RMP and AMP prevalence rates per year and shopper and media profile

	Total	AMP%			RMP%		
		2019	2020	Total	2019	2020	Total
		897	895	1792	897	895	1792
Online shop	No	16%	32%*	23%	11%	21%*	16%
	Yes	37%	58%*	48%	30%	51%*	41%
Night shop	No	21%	40%*	30%	15%	31%*	23%
	Yes	52%	71%*	62%	42%	63%*	53%
Vending/automated shop	No	21%	41%*	31%	16%	32%*	24%
	Yes	48%	69%*	58%	39%	60%*	50%
Newsagent's shop	No	20%	46%*	34%	14%	36%*	25%
	Yes	32%	46%*	39%	26%	39%*	32%
Petrol station shop	No	21%	38%*	29%	15%	28%*	22%
	Yes	39%	66%*	52%	32%	59%*	45%
GIBT grouped	Low	15%	27%*	21%	11%	22%*	17%
	2	18%	37%*	27%	12%	29%*	20%
	3	24%	40%*	32%	20%	34%*	27%
	4	30%	55%*	41%	22%	43%*	32%
	High	46%	69%*	59%	36%	56%*	47%
Internet browsing	No	20%	51%*	35%	18%	43%*	30%
	Yes	28%	45%*	36%	20%	36%*	28%
TV	No	29%	57%*	44%	23%	46%*	35%
	Yes	25%	42%*	33%	19%	34%*	26%
Radio	No	28%	47%*	38%	23%	39%*	31%
	Yes	24%	45%*	35%	17%	35%*	26%
Newspaper	No	27%	46%*	36%	20%	37%*	28%
	Yes	23%	46%*	35%	20%	36%*	29%
Magazine	No	26%	46%*	36%	20%	37%*	28%
	Yes	27%	47%*	40%	22%	39%*	33%
Newspaper digital	No	26%	45%*	36%	20%	37%*	29%
	Yes	26%	48%*	37%	19%	38%*	28%
Magazine digital	No	26%	45%*	35%	19%	36%*	28%
	Yes	32%	57%*	46%	27%	46%*	37%
Facebook	No	16%	39%*	27%	11%	30%*	20%
	Yes	32%	50%*	42%	25%	41%*	34%
Twitter	No	23%	43%*	33%	17%	35%*	26%
	Yes	52%	72%*	62%	44%	60%*	53%
Instagram	No	20%	38%*	29%	15%	30%*	22%
	Yes	56%	72%*	65%	46%	62%*	55%
YouTube	No	22%	40%*	31%	16%	32%*	24%
	Yes	49%	69%*	60%	41%	57%*	50%

Chi-Square Tests 2020–2019*p < 0.05

The significant chi-squares values for most group*period cross-tabulations in Tables 2, and 3 indicate that overall, the RMP and AMP prevalence of mobile payment increased significantly during the corona crisis amongst almost all groups under investigation. Exception to the rule are the AMP scores for the youngest age group, students and independent profession showing no significant increase.

### Individual differences in M-payment

Chi-square analyses were run to examine significant differences per sociodemographic grouping, reported in Table 2. Except for the regional difference between the Dutch and French, the significant differences between groups on the total sample applying to the RMP scores are like those for the AMP scores. While the Flemish part (38%) reported a higher AMP rate than the French part (33%), both regions report an equal portion of regular users. No differences could be found for urbanization, gender, or single status overall or per year.

The included economic profile background variables show a significant relationship with RMP and AMP scores in the expected direction. We find a higher occurrence of mobile payment among those with a higher education level, a higher household income, and working versus those with lower education, income, and not working. Except for students, we find lower mobile payment scores among blue-collar, unemployed, and home caregivers, with a higher score for white-collar workers. A strong age effect shows that adoption and regular use are still mainly occurring amongst the younger population.

In Table 3, we report the prevalence of AMP and RMP in the different shoppers and media variables available. Based on Chi-square analyses, all social media measures show a significant positive association with RMP and AMP. Furthermore, a significant positive association could be observed for digital magazine readers, while TV and radio audiences show a significant negative association between RMP and TV with AMP. No significance could be identified for internet browsing, paper/digital newspapers, and paper magazine audiences with AMP or RMP or radio with AMP. All convenience channels exhibit a significant association with RMP and AMP, and the general impulsive buying tendency score shows a significant positive association (The GIBT was grouped in 5 n-tiles for reporting purposes in Table 3). Given the parallels between RMP and AMP, we report further on RMP in the context of this paper.

Logistic regression was run on the RMP score, the available socio-demographic, media usage, retail patronage variables, and the period indicator as determinants to understand individual differences. Given some small sub-group sizes in the professional/occupational profile, the Social Grade Composite Score (SGCS) was used. Multicollinearity was checked using the procedure described by Midi, Sakar, and Rana [81], using a randomized normal variable as a dependent variable. Some apparent correlations exist between age and professional profile, resulting in higher than acceptable collinearity diagnostics. Pre-pensioners are all in the category above 54 (r = 0.90), while students can be found entirely in the youngest group below 25 (r = 0.95). The youngest age group and students also showed a VIF value above 2.5 which may cause concern. Including the SGCS score, no VIF value was higher than three, and further collinearity diagnostics show no condition index is higher than 15. None of the variance proportions of each regression coefficient is strongly associated with any dimension, so multicollinearity should not pose a significant problem further in the analysis.

Analysing the stepwise procedure's output in Table 4, the stepwise model shows an appropriate fit according to the Hosmer and Lemeshow test. Final Nagelkerke R Square as pseudo-indicator for explained variance reached 31%. The periodic effect between 2019 and 2020 is apparent, with an odds ratio running up to 2.54 (CI95: (1.99–3.24)) in the last step confirming hypothesis one. Also, the age effect can be observed with an odds ratio of 0.98 (CI95:(0.97–0.99)) with increasing age confirming hypothesis two. The lower chance for M-payment in the French-speaking part is apparent with an odds ratio of 0.75 (CI95: (0.58–0.98)). The SGCS as a social-economic factor shows a significant positive effect (Exp(B) = 1.13, CI95: (1.06–1.21)), supporting hypothesis six. In the first stage, we introduced the socio-demographic variable from a digital divide point of view. As such, we also noted significantly lower odds for women (Exp(B) = 0.68, CI95: (0.54–0.86)) and higher odds for those with kids (Exp(B) = 1.35, CI95: (1.06–1.70)), but these effects did not withstand in a concurrent model when introducing underlying shopper profiles. Also, the odds between singles or those living together show no differences. Also, income levels do not show any significant difference within our sample. We have to reject hypotheses two, four, five, and seven.

**Table 4:** Logistic regression: odds-ratios on regular mobile payment users (RMP)

Exp (B) (95% CI)	Step 1	Step 2	Step 3
Year (2020)	2.57 [2.05–3.22]*	2.62 [2.06–3.33]*	2.54 [1.99–3.24]*
Region (FR)	0.80 [0.64–1.01]	0.72 [0.56–0.91]*	0.75 [0.58–0.98]*
Urban (Y)	1.11 [0.89–1.39]	1.00 [0.78–1.26]	0.97 [0.76–1.24]
Sex (F)	0.68 [0.54–0.86]*	0.90 [0.70–1.15]	0.86 [0.67–1.11]
Age	0.95 [0.94–0.96]*	0.97 [0.96–0.98]*	0.98 [0.97–0.99]*
Single (Y)	0.82 [0.64–1.04]	0.91 [0.70–1.18]	0.85 [0.65–1.12]
Kids (Y)	1.35 [1.06–1.70]*	1.14 [0.89–1.46]	1.14 [0.88–1.46]
SGCS	1.11 [1.04–1.18]*	1.12 [1.05–1.20]*	1.13 [1.06–1.21]*
Income level	1.05 [0.92–1.20]	0.99 [0.86–1.15]	0.98 [0.84–1.14]
GIBT		1.04 [1.02–1.05]*	1.03 [1.01–1.05]*
Online shop (Y)		2.27 [1.76–2.92]*	2.20 [1.70–2.85]*
Night outlet shop (Y)		1.52 [1.09–2.12]*	1.38 [0.98–1.95]
Vending shop (Y)		1.31 [0.96–1.81]	1.28 [0.92–1.77]
Newsagents shop (Y)		0.94 [0.73–1.22]	0.92 [0.70–1.20]
Petrol station shop (Y)		1.82 [1.36–2.43]*	1.82 [1.35–2.44]*
Facebook (Y)			1.38 [1.03–1.84]*
Twitter (Y)			0.83 [0.53–1.32]
Instagram (Y)			1.93 [1.34–2.78]*
YouTube (Y)			1.02 [0.71–1.47]
Internet browsing (Y)			0.88 [0.62–1.25]
TV (Y)			0.91 [0.65–1.26]
Radio (Y)			0.95 [0.72–1.26]
Newspaper (Y)			1.36 [0.93–1.98]
Magazine (Y)			0.69 [0.39–1.22]
Newspaper digital (Y)			0.95 [0.71–1.27]
Magazine digital (Y)			0.95 [0.57–1.58]
Step *χ*^2^	247*	163*	25*
Model *χ*^2^	247*	**410**	**435**
*Df*	**9**	**15**	**26**
Nagelkerke *R*^2^	**0.19**	**0.29**	**0.31**
Hosmer & Lemeshow Test	**8.64**	**7.31**	**10.3**

^*^p < 0.05, *SGCS* Social grade composite score, *GIBT* General Impulse buying tendency

With regards to the shopper profile, the odds increase with general impulse buying tendency (GIBT) (Exp(B) = 1.03, CI95: (1.01–1.05)) and amongst Internet/Online shoppers (Exp(B) = 2.20, CI95:(1.70–2.85)) confirming hypotheses eight and nine. With regards to the convenience channels, only fuel station patronage shows a significant effect (Exp(B) = 1.82, CI95:(1.35–2.44)). Night outlet shoppers (Exp(B) = 1.52, CI95:(1.09–2.12)) showed a higher odds ratio in step 2, but this effect disappeared by introducing social media access. Hence, we can only accept hypothesis 10. Further observation shows that the odds for Facebook users (Exp(B) = 1.38, CI95:1.03–1.84), and Instagram users (Exp(B) = 1.93, CI95:1.34–2.78) are significantly higher. None of the other channel's audiences reaches significance. So also, hypothesis eleven can only be partially accepted.

## Discussion

Our data provide empirical academic evidence from a user point of view that the COVID-19 crisis accelerated the adoption of new retail technologies like mobile payment with an apparent positive periodic effect between 2019 and 2020 for both adoption (AMP) and more regular use (RMP) of mobile payment. It remains to be seen whether such a shift is sustained in a post-pandemic world and becomes habitual and widespread. The data in this study provided a unique opportunity to follow up on this prominent issue. The odds of being a regular user were 2.5 times higher in 2020 versus 2019. Given the current societal trends, one could expect mobile payment to mature in the coming years and become a dominant payment method. Given such a trend, it is paramount for retailers to offer this method both offline and online, as Belgium seems to move further towards a cashless society. The observed trend seems to support the previous mentioned industry report’s findings for Belgium [36, 65].

Considering our second research question, we further analysed socio-demographic differences. For the central part, differences observed during the crisis were already established before the crisis. The increase in mobile payment adoption can be observed in most layers of society, except for the youngest age group, which was already higher in 2019, and amongst the unemployed, which at a low level shows no significant increase. The initial gender effect with lower odds for women and the kid's effect showing higher odds for those with kids seems to disappear once controlled for underlying shop-related profiles. This could explain the inconclusive results of previous studies regarding the effect of gender, which did not control for such variables.

Our hypotheses regarding age and social grade effect remained valid in concurrence with other profile variables showing a higher occurrence of mobile payment among younger ages and higher socioeconomic profiles. The age effect is in line with previous American and European findings. Previous results on socioeconomic variables including income, education, working and occupational status were inconclusive. Our research findings indicate no relationship with income level but rather a higher odd with a higher social grade status.

Regional differences in Belgium are usually explained by the difference in socioeconomic profile of its citizen. Given the regional difference remains after controlling for socio-economic profile indicates that other regional cultural differences like Hofstede’s cultural dimensions, could be responsible for the difference in the adoption of mobile payment in both regions. Previous research reported important cultural differences between Flanders (Dutch) and Wallonia (French) on Hofstede’s cultural dimensions, which were also reflected in their respective e-commerce websites [82].

Our third research question is related to the retail context. We observe a clear association between general impulse buying tendency and mobile payment. The link between internet/online shopping and mobile payment is firmly established. This robust finding confirms previous research hypothesizing impulse buying and mobile payment to be related [69].

Our data seem to only partially support the hypotheses that the adoption and use of mobile payment might be linked to all types of convenience stores. A higher incidence of mobile payment among petrol station shoppers was found, but not for vending, newsagent, or night outlet shoppers.

Our findings support the notion that mobile payment adoption varies across different economic, cultural, social, and retail context [14].

In relation to our fourth research question, we assumed social media to be an indicator of mobile payment. However, this does not seem to apply similarly to all social network sites. Social media users of Facebook and Instagram, in particular, seem to be open to mobile payments. These channels facilitate social selling with specific shop-button features, allowing people to shop easily from the brand's photos and videos they encounter. Furthermore, a closer look at our data revealed a clear association between general impulse buying tendency and mobile payment and between mobile payment and Facebook and Instagram users. Not surprisingly, Aragoncillo and Orús [83] pointed out that general impulse buying is related to these social network sites. Furthermore, social network sites like Facebook have been identified as “Superbrands” that evoke trust during the pandemic [84]. Younger ages, general impulse buying, and Instagram and Facebook users are related. Nonetheless, they all seem to have a significant unique contribution in a concurrent model.

## Implications

### Policy

With such a sudden growth in adoption, it can be expected that not everyone has the necessary digital and financial competencies to use mobile payment rationally or is aware of the potential risks. So far, the hypothesis that mobile payment is associated with more problematic financial behavior was not supported in a European context but should be closely watched by policymakers. Our findings support the idea of considering financial vulnerability [12], given the inequality in adoption and usage, is less related to access but more to education and age.

So, from a digital divide and policy perspective, the government should be aware of this trend to ensure that the necessary framework is available to allow the technology to grow by providing necessary legislation, infrastructure, and support. This must safeguard that everyone can rely on reliable and safe financial payment services making the transition towards a cashless society [12], Furthermore, the government is well advised to communicate in a segmented approach. In terms of closing the digital divide gap and ensuring financial inclusion they need to focus on the profile of none-users, meaning older and less educated people. For informational and educational purposes, they can target users and heavy users. Social media seems an appropriate channel to reach them. Investing in financial education is advisable for all.

### Retail

Retailers should be aware of their client's profiles. Especially when targeting a younger audience or being situated in a higher upmarket neighbourhood, they should enable mobile payment options. The fact that mobile payments for impulse purchases may be more readily used indicates that retailers are commercially well advised to offer mobile payments as an incentive to facilitate such transactions not only online. Given the higher adoption amongst online shoppers, it is also paramount within an omnichannel strategy to offer mobile payment options.

For retailers looking for an omnichannel strategy or who want to increase their efforts in social selling, mobile payment is not even an option; it is a prerequisite. They need to be where their target audience is. It could be the opposite for financial service providers bringing mobile payments to market. If they want to attract first-time users, they could select those channels where they find the most nonusers. Interestingly, a recent study for a specific online retailer showed that the profile online shoppers most attracted by social media marketing were females, cash customers, and the less affluent socioeconomic groups. Retailers to which such a profile applies are well advised to provide the most convenient payment method [85]. If a digital strategy is rolled out, retailers need to consider the characteristics and attributes of the social media platform to be consistent with what the retailers try to achieve [86].

## Limitations and suggestions for future research

Given the limitation of a repeated cross-sectional approach, further research could opt for a longitudinal approach to capture trends in adopting new retail technologies like mobile payment after the COVID-19 pandemic. Our primary variable only captures the frequency of use, while researchers could also investigate the number of purchases, type of purchases, and the amount paid via mobile payment. Given the limitation of our sample to 18 to 65-year-olds, further research can also look at older seniors and younger people.

Our current finding gives further guidance and support where the field of technology acceptance should be heading, considering a maturing mobile payment market. In line with previous research [7, 10, 11, 16, 20], researchers need to be aware that with a maturing mobile payment market that; (1) earlier findings might not apply anymore and could be time specific, (2) should focus on differences within mobile payment options, (3) should switch from intentions to actual use or continuance use as the primary dependent variable, (4) could start to investigate preference or market/usage share as the main variable of interest, (5) differentiation in a mature market is not only a question of superior technology or product but also of branding.

Researchers investigating technology acceptance should be clear that they need to apply moderator variables to inform the field better, both technology providers and retailers, about what kind of features the user prefers. Both ages and socioeconomic-related moderators should be prime candidates to be incorporated within technology acceptance models investigating mobile payment. The need for more research and investigation into the role of trust and risk within specific target groups (ages,socio-economic profiles) and how they influence intentions and actual usage could advise policymakers further [87]. Researchers could further investigate whether the differences observed in age and education might be caused by underlying factors like knowledge and resistance toward recent technology.

Besides background variables like socioeconomic profile and age, the observed differences in our data warrant further investigation into specific shopper orientation-related differences like general impulse buying tendency as moderators in the field of mobile payment using technological acceptance models. Future research can investigate which factors in such models, like the perceived utility and ease of use, might be moderated. The differences between social media users also warrant further investigation into the role of social media in adoption models. Mobile payment adoption was higher for internet/online and petrol station shoppers, while not for other convenience channels. The perceived utility and ease of use might be considered higher amongst the former channels, which could be further investigated.
